# Challenges of Biomarker Application in Patients with Cardiorenal Syndrome

**DOI:** 10.3390/biomedicines14081864

**Published:** 2026-08-20

**Authors:** Elina Khattab, Sotiris Kyriakou, Dimitris Karelas, Maria Ioannou, Evangelos Tatsis, Panagiotis Bouzios, Andreas Mitsis, Constantinos H. Papadopoulos, Nikolaos P. E. Kadoglou

**Affiliations:** 1 Medical School, University of Cyprus, 215/6 Old Road Nicosia-Limassol, Nicosia 2029, Cyprus; khattab_elina@outlook.com; 2 Barts and The London School of Medicine and Dentistry, London EC1M 6BQ, UK; s.kyriakou@nhs.net (S.K.); bouzios3004@gmail.com (P.B.); 3 2nd Department of Cardiology, Red Cross General Hospital of Athens, 11526 Athens, Greece; dim.f.karelas@gmail.com (D.K.); tatsisevangelos@gmail.com (E.T.); papcost@gmail.com (C.H.P.); 4 Department of Cardiology, Limassol General Hospital, Limassol 4131, Cyprus; mar.ioannou25@gmail.com; 5 Department of Cardiology, Nicosia General Hospital, 215 Old Road Nicosia-Limassol, Nicosia 2029, Cyprus; andymits7@gmail.com

**Keywords:** cardiorenal syndrome, troponin, natriuretic peptides, cystatin C, interleukins, sodium-glucose cotransporter-2 inhibitors (SGLT2i)

## Abstract

**Background/Objectives:** Cardiorenal syndrome (CRS) is associated with substantially higher morbidity and mortality than either isolated cardiac or renal dysfunction. The application of classical and novel biomarkers has been tested in prompt diagnosis and monitoring of patients with CRS. **Methods:** This is a comprehensive literature review following a structured approach. We searched MEDLINE and Embase databases from January 2000 to December 2025. **Results:** The pathophysiology of CRS is complex, and the present review attempts to shed light on the clinical interpretation of the most widely used biomarkers as indices of diagnosis and prognosis. Among them, troponin is elevated in CRS and its absolute levels retain prognostic value, while changes in its levels over time may assist in the diagnosis of acute coronary syndrome. Natriuretic peptides are highly influenced by coexistence of chronic kidney disease (CKD) and in this context have considerable diagnostic and prognostic value. The combination of cystatin C, a biomarker of renal dysfunction, with cardiac biomarkers may create a powerful risk algorithm. Most recently, gene profiling and proteomics have the potential to stratify patients with CRS; however, more data from large cohorts are required for their validation. The therapeutic modulation of biomarkers in CRS patients may help elucidate the underlying pathophysiologic mechanisms. Sodium-glucose cotransporter-2 inhibitors (SGLT2i) have emerged as first-line therapy for patients with CRS, despite the fact their mechanisms are mostly unknown. Significant changes in the aforementioned biomarkers and the inflammatory factors may explain their emerging beneficial effects on both heart failure and CKD. **Conclusions:** The use of biomarkers has increased rapidly in recent years for diagnosis, surveillance and prognostic stratification in CRS.

## 1. Introduction

Cardiorenal syndrome (CRS), driven by the growing burden of heart failure (HF) and chronic kidney disease (CKD) in ageing populations, has become a clinically significant entity and is associated with substantially higher morbidity and mortality than either isolated cardiac or renal dysfunction [1,2]. It does not simply represent the coexistence of HF and CKD but rather reflects complex interactions among haemodynamic, neurohormonal, inflammatory and metabolic disturbances which perpetuate bidirectional organ injury.

CRS categorization is based on the initial organ affected and the timeframe in which renal impairment occurs relative to HF. Type 1 is known as “acute CRS” and refers to the development of acute kidney injury (AKI) caused by an acute decline in cardiac function. Type 2 is “chronic CRS”, which involves long-standing cardiac dysfunction that ultimately results in CKD. The reverse sequence occurs in “acute reno-cardiac syndrome” (type 3) when AKI causes acute cardiac dysfunction, and “chronic reno-cardiac syndrome” (type 4) represents the long-term cardiovascular effects of CKD. “Secondary CRS” (type 5) includes all systemic diseases (e.g., sepsis, diabetes mellitus) that can concurrently affect both cardiac and renal functions [1,2,3,4].

While categorizing these subtypes provides clinicians with a useful paradigm for organizing their approach to diagnosis and treatment, overlap among the subtypes is common. This clinical heterogeneity has several implications for biomarker use in CRS, as different subtypes may arise from overlapping yet distinct pathophysiological mechanisms. Accordingly, each biomarker must be interpreted within the context of the patient’s clinical presentation.

Natriuretic peptides (NPs), such as B-type natriuretic peptide (BNP) and N-terminal pro–B-type natriuretic peptide (NT-proBNP), reflect myocardial wall stress whereas cardiac troponins (cTn) indicate myocardial injury [5]. Both serum creatinine and serum cystatin-C can be used to estimate glomerular filtration rate (eGFR), although neither specifically assesses structural kidney injury [6,7,8]. In the presence of suspected renal damage, biomarkers of acute tubular injury, such as neutrophil gelatinase-associated lipocalin (NGAL) and kidney injury molecule-1 (KIM-1), may be considered [9,10]. Biomarkers of inflammatory activity, including interleukins (ILs) and tumour necrosis factor-α (TNF-α), help characterize the inflammatory component of CRS, an important aspect of disease progression [11]. Galectin-3 (gal-3) and soluble Suppression of Tumorigenicity-2 (sST2) are associated with fibrotic and remodeling pathways in CRS [12]. Furthermore, ongoing integration of genomic and multi-omic approaches may clarify the molecular heterogeneity of CRS and support more precise biomarker-guided strategies [13,14].

Despite considerable advances, no single biomarker fully captures the complexity of CRS, and CRS-specific thresholds and true bidirectional specificity remain undefined. Reduced eGFR, for example, may lead to accumulation of several cardiac biomarkers, rendering diagnostic cut-offs derived from HF populations without advanced CKD potentially unreliable in this setting [15,16,17,18]. Serial assessment and consideration of the acute or chronic CRS phenotype therefore provide greater clinical utility [15]. Current research has evaluated biomarkers individually or within HF or CKD populations, often without adequately accounting for renal confounding [16,17]. Consequently, structured frameworks for biomarker interpretation that incorporate renal function, temporal dynamics, and multi-marker approaches remain underdeveloped, and robust prognostic tools to guide clinical decision-making in CRS are still lacking [15,16,17,18].

This comprehensive review synthesizes the current clinical evidence underpinning the pathophysiology, diagnostic performance, and prognostic relevance of biomarkers in CRS, and outlines a structured framework for their context-specific interpretation across the spectrum of CRS subtypes.

## 2. Search Strategy and Study Selection

**Figure 1:**
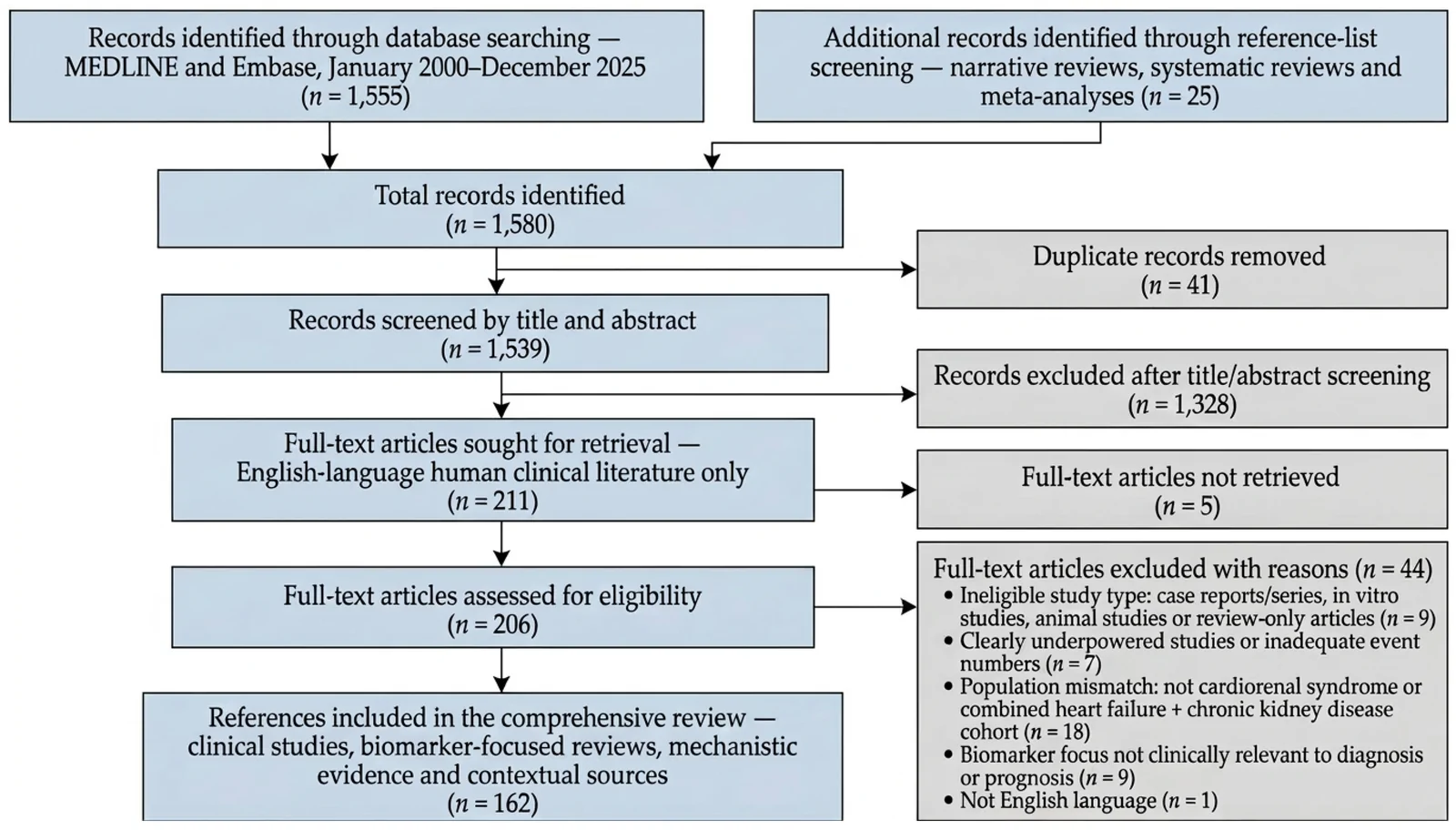
Flow diagram of the structured literature search and study-selection process. MEDLINE and Embase were searched from January 2000 to December 2025 and were supplemented by reference-list screening of relevant narrative reviews, systematic reviews and meta-analyses. After duplicate removal, title/abstract screening and full-text eligibility assessment, 162 clinical, biomarker-focused, mechanistic and contextual sources were included in the comprehensive review.

**Figure 2:**
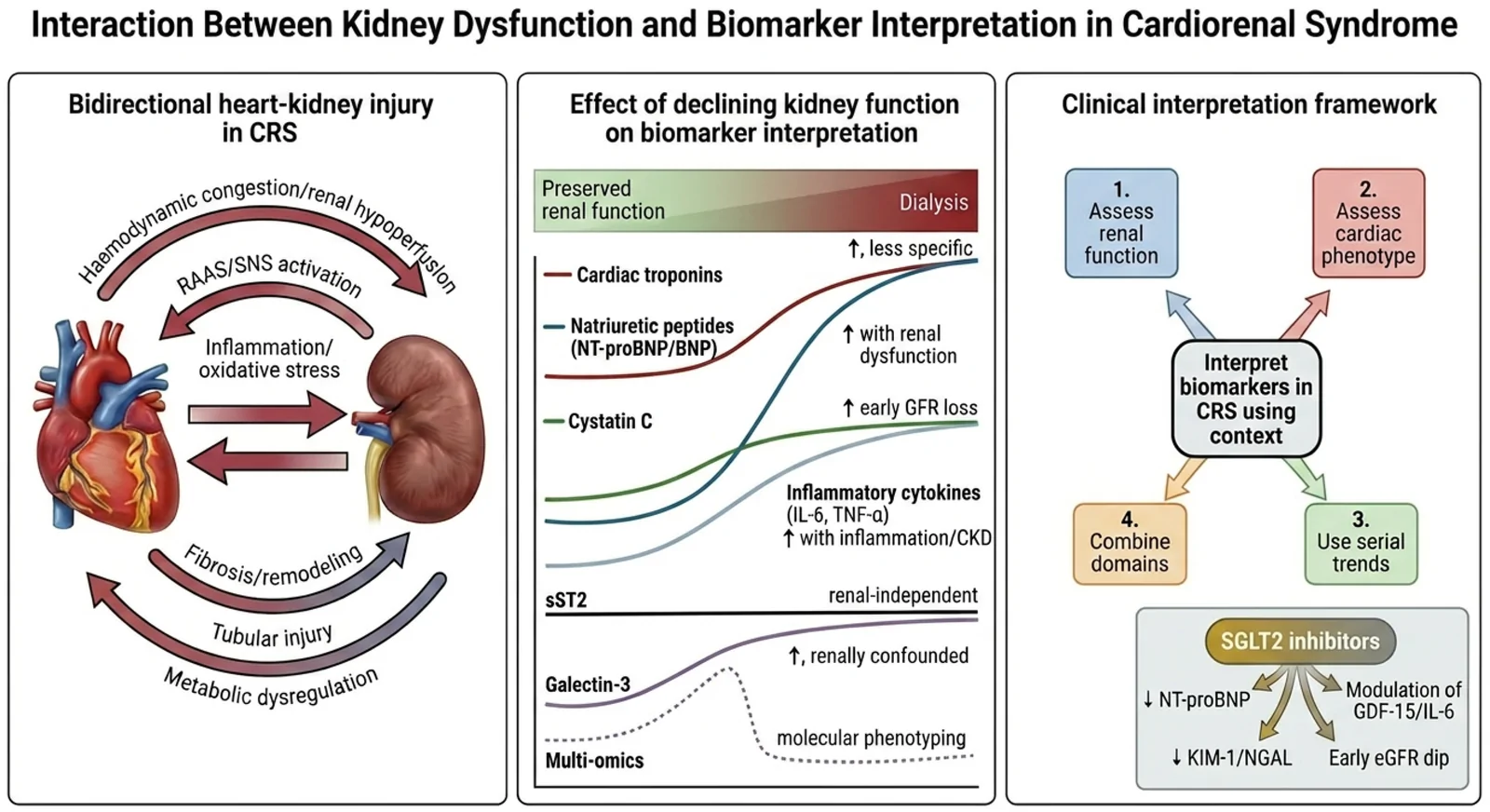
Interaction between kidney dysfunction and biomarker interpretation in cardiorenal syndrome. The figure summarizes bidirectional heart–kidney injury, the effect of declining renal function on key biomarker groups, and a practical four-step framework for contextual biomarker interpretation in CRS.

**Figure 3:**
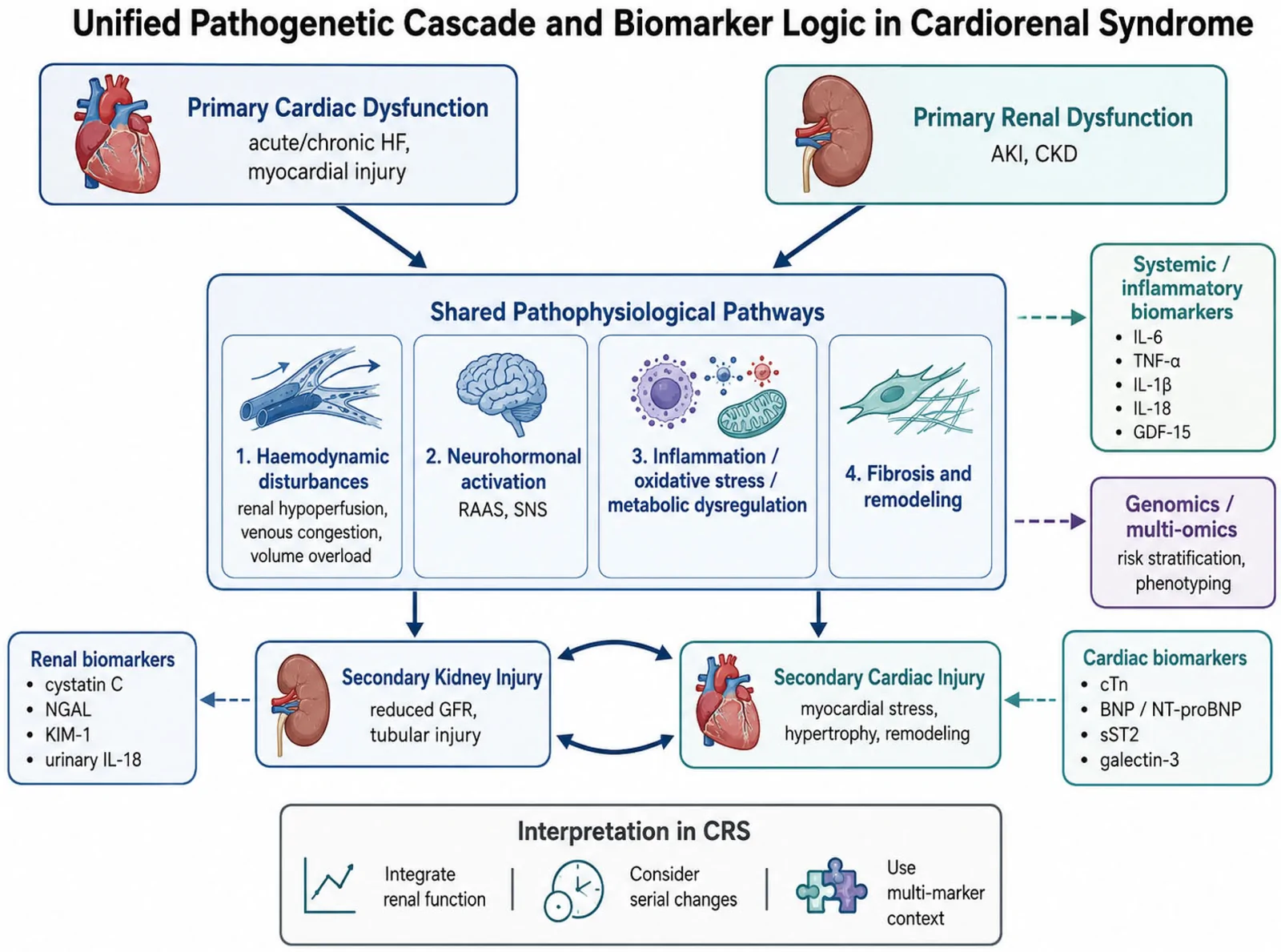
Unified pathogenetic cascade and biomarker logic in cardiorenal syndrome. The figure illustrates how primary cardiac or renal dysfunction may initiate a self-perpetuating cascade of haemodynamics, neurohormonal, inflammatory, oxidative, metabolic, and fibrotic disturbances, leading to secondary injury in the other organ. It also summarizes the principal biomarker groups involved in this process, including cardiac biomarkers, renal biomarkers, systemic inflammatory biomarkers, and genomics/multi-omics approaches, and highlights the need to interpret biomarkers in the context of renal function, serial changes, and a multi-marker framework.

This is a comprehensive literature review and for this purpose we followed a structured search approach. We searched MEDLINE and Embase databases from January 2000 to December 2025. Except for case studies and preclinical studies (in vitro and animal), we included all other types of clinical studies (observational, randomized, non-randomized, prospective, retrospective). In addition to this, we meticulously looked at previous review articles (narrative reviews, systematic reviews, and meta-analyses) to identify additional relevant references for inclusion. All included studies were published in English. Small, underpowered studies were excluded. The study-selection process is summarised in Figure 1. We used the following search terms, including Medical Subject Headings: cardiorenal syndrome (CRS); heart failure (HF); kidney or renal failure; chronic kidney disease; dialysis; biomarkers; natriuretic peptides; cystatin; metabolomics; proteomics; cytokines; inflammatory biomarkers; interleukins; galectin-3; diagnosis; prognosis; therapy. Studies were eligible only if they included cohorts with either CRS or concomitant HF and CKD. OpenAI ChatGPT, GPT-5.6(OpenAI, San Francisco, CA, USA) was used to assist in the initial visual development of Figure 2 and Figure 3. The figures were subsequently reviewed and revised by the authors to ensure that all scientific content was consistent with the evidence presented in the manuscript. Generative artificial intelligence was not used for the literature search, study selection, evidence extraction, data analysis, or interpretation of the reviewed evidence.

## 3. Biomarkers in CRS

The clinical application and interpretation of biomarkers in CRS are summarized in Table 1.

The interaction between kidney dysfunction, biomarker interpretation and clinical decision-making in CRS is conceptually illustrated in Figure 2.

Abbreviations: BNP, B-type natriuretic peptide; CKD, chronic kidney disease; CRS, cardiorenal syndrome; GFR, glomerular filtration rate; NT-proBNP, N-terminal pro–B-type natriuretic peptide; RAAS, renin–angiotensin–aldosterone system; SNS, sympathetic nervous system; sST2, soluble suppression of tumorigenicity-2. From a pathogenetic perspective, CRS may be viewed as a self-perpetuating cascade in which primary dysfunction of the heart or kidney initiates haemodynamic, neurohormonal, inflammatory, and metabolic disturbances that subsequently promote injury in the other organ (Figure 3) [1,2,3,4,7,8]. In acute or chronic HF, renal hypoperfusion and venous congestion may lead to early renal dysfunction, which may be reflected by biomarkers such as cystatin C and urinary IL-18 [19,20,21]. Conversely, CKD may promote chronic myocardial injury, left ventricular hypertrophy, and volume overload, contributing to persistent elevations in cardiac troponins and NPs [18,22,23,24,25]. In parallel, systemic inflammatory and fibrotic pathways involving IL-6, TNF-α, sST2, and galectin-3 (gal-3) may amplify bidirectional organ injury, helping explain why multiple biomarker groups may be simultaneously altered in CRS [26,27,28,29,30,31].

Abbreviations: AKI, acute kidney injury; BNP, B-type natriuretic peptide; CKD, chronic kidney disease; CRS, cardiorenal syndrome; cTn, cardiac troponin; GDF-15, growth differentiation factor 15; GFR, glomerular filtration rate; HF, heart failure; IL-1β, interleukin-1β; IL-6, interleukin-6; IL-18, interleukin-18; KIM-1, kidney injury molecule-1; NGAL, neutrophil gelatinase-associated lipocalin; NT-proBNP, N-terminal pro–B-type natriuretic peptide; RAAS, renin–angiotensin–aldosterone system; SNS, sympathetic nervous system; sST2, soluble suppression of tumorigenicity-2; TNF-α, tumour necrosis factor-α.

### 3.1. Cardiac Troponin

#### 3.1.1. Cardiac Pathophysiology

Cardiac troponin I (cTnI) and cardiac troponin T (cTnT) are highly specific proteins of the cardiac contractile apparatus, involved in the regulation of contraction and relaxation of cardiomyocytes [16]. In the setting of cardiomyocyte injury, like myocardial ischaemia, mechanical stress, or inflammatory conditions, disruption of cellular integrity leads to the release of cTnI and cTnT into the circulation [17].

In patients with CKD, a persistent mild elevation of serum cTn concentration is frequently observed even when myocardial ischaemia is absent. Experimental evidence in CRS suggests that cardiomyocytes undergoing necrosis, and potentially apoptosis, exhibit loss of sarcolemmal integrity, resulting in the release of cTn into the circulation [18,22]. Several pathophysiological mechanisms have been proposed, including increased cTn release secondary to chronic subclinical myocardial injury and reduced renal elimination. The former mechanism seems to play a substantial role in the persistent elevation of cTnin patients with CRS [22,23]. Another characteristic of CKD patients is the left ventricular hypertrophy, due to increased mechanical wall stress, and adverse cardiac remodeling. It has been hypothesized that this may be the result of disturbances in mineral metabolism—particularly dysregulation of calcium and phosphorus, along with their regulators such as vitamin D, parathormone (PTH), and fibroblast growth factor 23 (FGF23). Additionally, uraemic toxicity, oxidative stress, and chronic low-grade inflammation associated with advanced CKD may contribute to persistent subclinical injury, ultimately leading to cTn elevation [18,24].

#### 3.1.2. Interaction with Renal Function

cTn is routinely used in clinical practice for the diagnosis of acute coronary syndromes (ACS). Similarly, in patients presenting with acute heart failure (AHF) without ACS, cTn levels are frequently elevated. Such elevations may also occur in individuals with renal dysfunction even in the absence of AHF or ACS. That makes the clinical interpretation of cTn elevation complex in CRS [24].

hs-cTnT concentrations correlate independently with eGFR and urinary albumin excretion, showing a progressive increase as renal function declines [18]. In patients with non-dialysis CKD without acute myocardial infarction (AMI) or HF, circulating hs-cTnT levels are frequently elevated compared with the healthy general population, with approximately 43–68% exceeding the 99th percentile upper reference limit [19,32]. Further data analysis provided a model demonstrating that, in patients with CKD, the threshold for the 99th percentile of serum hsTnT concentrations increases by 44% for each 15 mL/min/1.73 m^2^ decrease in eGFR [32]. A previous study examined how cTn levels change over time in pre-dialysis CKD. Chesnaye et al. followed 176 individuals for 24 months and found an average increase of 16% per year in cTn levels [33]. On the other hand, there are conflicting results regarding the impact of haemodialysis on cTn levels. Although some studies have demonstrated a significant reduction in cTn levels between pre- and post-dialysis measurements on average, the majority report that some patients exhibit an increase in cTn levels following dialysis. Tarapan et al. reported that higher blood flow rates and higher haemoglobin concentrations were independently associated with intradialytic increases in cTn levels, suggesting the possibility of silent myocardial injury during haemodialysis [34].

Beyond renal-related myocardial injury, cTn levels in CKD patients likely reflect underlying heart disease in the context of CKD, primarily left ventricular hypertrophy, subclinical systolic dysfunction, as evidenced by reduced global longitudinal strain, and, to a lesser extent, diastolic dysfunction and low-grade inflammation [35,36]. Persistently increased cTn levels in patients with renal impairment are associated with a higher risk of progression to overt cardiovascular disease, particularly HF and subsequent CRS, and may therefore have important prognostic value for hospitalizations and mortality.

#### 3.1.3. Diagnosis and Prognosis

In the acute setting, elevated hs-cTn levels in CKD patients presenting with chest pain remain highly sensitive for the diagnosis of AMI. However, specificity and overall diagnostic accuracy are reduced due to chronically elevated baseline concentrations. Therefore, serial measurements are essential to distinguish AMI from its chronic elevation due to myocardial injury. The measurement of cTn levels has also been recommended for mortality risk stratification in this population [37]. Higher cTn concentrations in CKD patients presented with AMI are associated with worse clinical outcomes and increased mortality risk [35]. Murphy et al. reported that, among patients with CKD presenting with AMI, both peak cTnT levels and eGFR were independent predictors of post-AMI AHF severity and mortality [38]. Additionally, elevated hs-cTnT concentrations have been identified as significant predictors of 2-year mortality in patients presenting with acute chest pain and impaired renal function (eGFR < 60 mL/min/1.73 m^2^), with an area under the curve (AUC) of 0.69, sensitivity of 74%, specificity of 63%, and a negative predictive value of 90% [32,39].

Among individuals hospitalized for AHF, the predictive power of hs-cTnT for all-cause mortality diminishes progressively as renal function worsens, particularly in patients with advanced CKD [16,40]. Nevertheless, a panel of four blood biomarkers—hs-cTnT, BNP, FGF23, and fibrinogen—has been proposed as a standalone score for predicting HF incidence among CKD patients [41].

Importantly, cTnT may help identify the coexistence of HF and CKD. Elevated cTnT levels (in addition to NGAL and Blood Urea Nitrogen) may suggest the presence of CRS, thereby supporting earlier diagnosis and more effective management [18]. A secondary analysis of the RELAX-AHF trial evaluated patients hospitalized with AHF and at least mild renal impairment (mean eGFR 53.3 mL/min/1.73 m^2^). The researchers examined the association of serial hs-cTnT levels with risk of renal failure or rehospitalization for HF within 60 days. The analysis demonstrated that greater increases in hs-cTnT concentrations over time were associated with greater likelihood of rehospitalization due to CRS [24]. According to the 2022 HF guidelines issued by the American Heart Association, the American College of Cardiology, and the Heart Failure Society of America, the addition of NT-proBNP or hs-cTnT to HF evaluation in adults with CKD reclassified nearly 20% of individuals into a higher HF stage, thereby improving risk discrimination [42]. In a meta-analysis of 9289 patients with chronic heart failure (CHF)—more than 5000 of whom had a baseline eGFR < 60 mL/min/1.73 m^2^—elevated hs-cTnI concentrations were linked to a higher risk of cardiovascular death and hospitalization. This association remained significant after adjusting for demographic factors, left ventricular systolic function, and NT-proBNP levels [24].

Overall, hs-cTn represents a key prognostic biomarker within the bidirectional interplay between cardiac and kidney diseases, predicting hospitalizations and mortality. However, hs-cTn interpretation in CRS is limited by assay-dependent differences, biological variability, and the absence of validated CRS-specific thresholds. Beyond renal dysfunction, left ventricular hypertrophy, underlying structural heart disease, AHF, systemic inflammation, and haemodialysis-related myocardial stress may increase troponin concentrations; therefore, serial changes should be interpreted within the full clinical context [17,22,24,34,35,36,37].

### 3.2. Natriuretic Peptides

#### 3.2.1. Cardiac Pathophysiology

NPs are strongly associated with HF severity and prognosis [22,37]. BNP and NT-proBNP are cardiac-derived peptides released primarily from the ventricular myocardium in response to myocardial stretch, increased intravascular volume, and neurohormonal activation. Circulating levels of both peptides are increased in patients with either chronic heart failure (CHF) or AHF. Within cardiomyocytes, BNP is initially synthesized as a 134–amino acid precursor (preproBNP), which is subsequently cleaved into a signal peptide and a 108–amino acid prohormone (proBNP). Upon secretion into the circulation, proBNP is enzymatically split in equimolar proportions into the biologically active BNP (32 amino acids), representing the C-terminal fragment, and the biologically inactive NT-proBNP (76 amino acids), corresponding to the N-terminal fragment [25,43]. NT-proBNP has a longer half-life than BNP (1–2 h depending on renal function versus 20 min) [44]. BNP is cleared by the natriuretic peptide receptors located in the kidney and vasculature and the neutral endopeptidase. Glomerular filtration and thereby renal excretion play only a minor role in the elimination of BNP. NT-proBNP can be filtered by the glomerulus and is only partially reabsorbed. Consequently, NT-proBNP can be detected in the urine of patients with AHF and has been suggested as a potential marker of cardiorenal injury [45].

NPs play a pivotal role in vascular homeostasis affecting both blood pressure (BP) and intravascular volume. For BP control, NPs promote vasodilation through the relaxation of vascular smooth muscle cells, the suppression of the renin–angiotensin–aldosterone system (RAAS), the inhibition of sympathetic tone, and the inhibition of endothelin-1 secretion. In addition, NPs regulate intravascular volume, as they influence electrolyte and fluid balance in the kidneys. They inhibit sodium reabsorption in both the proximal and distal nephron promoting natriuresis and diuresis. Through these combined effects, NPs decrease intravascular volume and BP. The consequent reduction in both cardiac preload and afterload may improve cardiac function especially in HF patients [43,46]. Collectively, the interplay between cardiac and kidney function shows a complex regulation of NPs in CRS.

#### 3.2.2. Interaction with Renal Function

Patients with HF and renal impairment, particularly when eGFR declines below 60 mL/min, tend to have higher concentrations of BNP and NT-proBNP. A number of studies have sought to elucidate their utility in this setting. In patients presenting with dyspnoea and impaired renal function, there is lower diagnostic accuracy for both biomarkers. Therefore, higher cut-off values are required to maximize sensitivity and specificity [24]. NT-proBNP values  >  450 pg/mL for patients aged  <  50 years (>900 pg/mL for patients between 50 and <75 years old and 1200 pg/mL for patients ≥ 75 years old) had a sensitivity of 85% and a specificity of 88% for diagnosing ADHF among subjects with eGFR  ≥  60 mL/min/1.73 m^2^ [25]. However, in patients with an eGFR < 60 mL/min/1.73 m^2^, there was a drop in specificity to 68%. Attempting to improve the specificity in these patients, the cut-off value was adjusted based upon the receiver-operating characteristic (ROC) curves to a single, age-independent value of 1200 pg/mL for patients with eGFR < 60 mL/min/1.73 m^2^. Applying that cut-off value, the researchers found increased sensitivity and specificity to 89% and 72%, respectively [22]. The investigators concluded that NT-proBNP testing, using modified cut-off values, is necessary for the evaluation of patients with suspected HF and renal dysfunction [25,37,47].

However, a pooled individual-level analysis of four large-scale randomized trials in patients with HF with mildly reduced or preserved ejection fraction (I-PRESERVE, TOPCAT, PARAGON, and DELIVER) demonstrated that the same NT-proBNP concentration was associated with a substantially higher absolute risk of adverse outcomes in individuals with reduced kidney function compared with those with preserved kidney function. These findings challenge proposals to adopt higher NT-proBNP reference ranges for patients with CKD and suggest that elevated NT-proBNP levels could be dissociated from reduced kidney function [48,49].

Overall, NT-proBNP testing is valuable in patients with CRS, and, at a given NT-proBNP level, the absolute risk of mortality is higher in patients with impaired renal function than in those with normal renal function [25].

#### 3.2.3. Diagnosis and Prognosis

The NPs have been extensively studied as biomarkers in patients with HF and CKD. Elevated NP concentrations are strong indicators of new-onset HF among patients with underlying kidney disease. In a cohort of 3483 individuals with CKD, participants in the highest NT-proBNP quartile (>433.0 pg/mL) had nearly a fivefold higher risk of incident HF compared with those in the lowest quartile (≤47.6 pg/mL). This relationship persisted after adjustment for renal function, medication use, and established cardiovascular risk factors [24].

Measuring elevated plasma concentrations of NPs in dialysis patients may be useful for identifying left ventricular hypertrophy, which can subsequently lead to cardiac dysfunction [49,50,51,52]. Conversely, higher NT-proBNP levels in HF populations have also been linked to a greater likelihood of requiring dialysis and to increased in-hospital mortality of individuals with moderate-to-severe AKI [37]. Among patients hospitalized for ADHF and concomitant CKD, NPs levels measured on admission may provide important prognostic information. A meta-analysis including over 2500 HF patients with impaired renal function demonstrated that elevated NT-proBNP on admission was associated with a 2.3-fold increased risk of mortality and/or major adverse cardiovascular events (MACEs) [24]. Other studies have shown that increased NT-proBNP levels on admission independently predicted 60-day mortality among patients with renal dysfunction. In addition, researchers found that discharge levels of NT-proBNP above the median were associated with mortality or readmission at 6 months [43].

In patients admitted with AHF and concomitant renal dysfunction, early changes in renal function—expressed as ΔeGFR%—have prognostic significance for long-term mortality, particularly among those with a limited decline in NT-proBNP (<30%) [53,54]. Pooling data from five prospective cohorts (1232 patients hospitalized for ADHF) confirmed that failure to achieve a reduction in NT-proBNP greater than 30% during hospitalization was associated with worse outcomes regarding all-cause mortality (HR 1.81; 95% CI 1.32–2.50) and the composite endpoint (HR 1.36; 95% CI 1.13–1.64). They were also 36% more likely to experience readmission or death within six months. In contrast, the worsening of renal function—irrespective of the degree—did not predict adverse outcomes independently from clinical variables and NT-proBNP reduction [24,55]. These findings suggest that effective decongestion in AHF, reflected by a meaningful decline in NPs levels, is a more powerful determinant of subsequent morbidity and mortality than transient renal deterioration, even in the context of type 1 CRS [24].

NPs are highly affected by the interaction between the heart and the kidneys. NP levels aid in identifying the presence and type of CRS and provide important prognostic information regarding disease severity and clinical outcomes. However, the interpretation of NPs in CRS is constrained by differences between BNP and NT-proBNP assays, intra-individual variability, and the absence of universally validated CRS-specific cut-offs. Beyond renal dysfunction, age, myocardial structure and function, neurohormonal activation, and changes in volume status during treatment may substantially influence their concentrations; therefore, serial trends should be interpreted alongside the patient’s congestion status and clinical phenotype [22,24,25,43,48,49].

### 3.3. Cystatin C

#### 3.3.1. Renal Pathophysiology

Cystatin C is a 13 kDa cysteine protease inhibitor produced at a constant rate by all nucleated cells, making it a useful marker for eGFR assessment. Unlike creatinine, cystatin C is less influenced by muscle mass, age, sex, and dietary protein intake, providing more accurate kidney function estimation in populations with altered body composition, including HF patients with cardiac cachexia or fluid overload [56]. In CRS, cystatin C serves as a sensitive marker of the pathophysiological interplay linking cardiac and renal dysfunction [57]. The cardiorenal axis involves haemodynamic perturbations, neurohormonal activation, inflammation, and oxidative stress that simultaneously affect both organs. Elevated cystatin C levels in CRS have also been associated with inflammatory markers including TNF-α, interleukin-6 (IL-6), and C-reactive protein (CRP), suggesting that it may reflect systemic inflammation in addition to impaired filtration [58].

#### 3.3.2. Interaction with Cardiac Function

The pathophysiological advantage of cystatin C in CRS lies in its ability to detect subclinical renal dysfunction before creatinine elevation. In acute decompensated HF, renal hypoperfusion and venous congestion may lead to early deterioration in renal function that cystatin C can detect earlier than creatinine [19]. This creatinine-blind period may represent a critical therapeutic window where interventions could prevent progression to established AKI.

#### 3.3.3. Diagnosis and Prognosis

Cystatin C demonstrates superior diagnostic performance compared to serum creatinine for detecting AKI in the setting of AHF. A rise in cystatin C greater than 0.3 mg/L within 48 h of admission has been associated with AKI and with worse in-hospital outcomes [19]. The diagnostic utility of cystatin C is particularly evident in the creatinine-blind GFR range of 60–90 mL/min/1.73 m^2^, where creatinine may remain within normal limits despite significant nephron loss [56]. In HF populations, cystatin C-based GFR equations (CKD-EPI cystatin C or combined creatinine-cystatin C equations) provide more accurate staging of CKD and better risk stratification than creatinine-based estimates alone [59]. Higher cystatin C levels generally reflect worse renal function, although specific diagnostic thresholds may vary according to the population and clinical setting [57]. The combination of elevated cystatin C with NPs (BNP or NT-proBNP) creates a dual-organ assessment strategy that simultaneously evaluates cardiac wall stress and renal filtration capacity, enabling comprehensive cardiorenal phenotyping at the bedside [57,60].

Cystatin C provides important prognostic information across the spectrum of CRS [61]. In AHF, elevated cystatin C levels (>1.30 mg/L) independently predict 12-month all-cause mortality after adjustment for established risk factors including age, ejection fraction, and natriuretic peptide levels [62]. This prognostic value persists in patients with preserved creatinine levels, identifying a high-risk subgroup that traditional renal markers may miss [59,62]. The combination of cystatin C with cardiac biomarkers creates powerful risk stratification tools. Patients with dual elevation of cystatin C and NT-proBNP demonstrate a markedly increased risk of MACEs compared to those with normal levels of both markers [60]. This combined approach identifies patients with established CRS who may benefit from intensified monitoring and therapeutic optimization [58]. Serial cystatin C measurements during HF hospitalization may also provide dynamic prognostic information. Patients with persistently elevated or rising cystatin C levels during treatment tend to have worse outcomes, although these changes should always be interpreted in the context of overall clinical status and degree of decongestion [60]. Overall, cystatin C appears to be a sensitive biomarker of renal dysfunction in CRS with clinically relevant applications in both diagnosis and risk stratification.

However, cystatin C is not a specific marker of structural kidney injury, and assay standardization and CRS-specific thresholds remain limited. Beyond renal function, systemic inflammation, thyroid dysfunction, corticosteroid treatment, smoking, and obesity may influence cystatin C concentrations, limiting its interpretation as a standalone biomarker [56,57,58].

### 3.4. NGAL and KIM-1

#### 3.4.1. Pathophysiology

Neutrophil gelatinase-associated lipocalin (NGAL) and kidney injury molecule-1 (KIM-1) are biomarkers of tubular injury. NGAL is released following tubular epithelial injury and may increase before serum creatinine, whereas KIM-1 is expressed in injured proximal tubular cells and reflects structural renal damage [6,9].

#### 3.4.2. Interaction with Renal Function

In patients with cardiorenal disease, increased NGAL and KIM-1 levels may indicate tubular injury that is not adequately reflected by serum creatinine or eGFR. In HFpEF, both biomarkers have been associated with mortality and HF hospitalization [63]. In CRS type 1, NGAL and KIM-1 may also increase alongside inflammatory and oxidative stress markers [28,64].

#### 3.4.3. Diagnosis and Prognosis

NGAL and KIM-1 may support the early identification and monitoring of tubular injury in CRS. Reductions in these biomarkers during treatment have been associated with improvement in cardiac and renal function [64,65,66,67]. However, neither biomarker is specific to CRS, and standardized CRS-specific thresholds are lacking. They should therefore be interpreted as complementary markers rather than isolated diagnostic tests.

### 3.5. Genomics and Multi-Omics Biomarkers

#### 3.5.1. Proteomic Profiling and Protein Biomarkers in Cardiorenal Disease

Proteomics has emerged as an important tool for elucidating molecular mechanisms underlying CRS, revealing inflammatory, vascular, and fibrotic pathways. Untargeted plasma proteomics using liquid chromatography–tandem mass spectrometry (LC-MS/MS) identified 2054 proteins across CKD stages. Of these, 333 were significantly altered in end-stage disease, including complement components C1r, C1s, and complement factor D, confirming activation of immune and coagulation pathways. Novel cardiovascular-related proteins such as lysozyme C and leucine-rich alpha-2-glycoprotein (LRG1) were inversely correlated with eGFR, and elevated LRG1 independently predicted mortality in haemodialysis patients, highlighting its prognostic and mechanistic relevance [13]. Similarly, aptamer-based proteomics showed that 67.3% of 1054 circulating proteins correlated with eGFR and were involved cardiovascular pathways such as angiogenesis, hypertension, and cardiac fibrosis, identifying CKD-associated cardiovascular biomarkers including fibroblast growth factor-7 and TNF receptor family proteins [14]. Large-scale proteomics in myocardial infarction (MI) and CKD cohorts further identified adrenomedullin, FABP4, GDF15, TNF-R1, TNF-R2, and TRAIL-2 as predictors of mortality and HF, reflecting inflammatory, apoptotic, and metabolic pathways [68]. Complementary analyses of 2920 proteins identified cardiovascular risk biomarkers including ADM, ANGPTL3, IGFBP4, MMP3, MMP12, and NPs, with proteomic risk scores outperforming clinical and polygenic models [69]. Machine learning-derived proteomic signatures based on 238 proteins predicted incident cardio-kidney-metabolic disease (HR 1.87 per SD), while a 16-protein risk score improved cardiovascular event prediction (AUC 0.77–0.80 vs. 0.57–0.72), implicating fibrosis, thrombosis, and vascular inflammation pathways [70,71].

Proteomic profiling has also identified key mediators and predictors of HF in CKD. Aptamer-based quantification of 1068 proteins identified angiopoietin-2, spondin-1, tartrate-resistant acid phosphatase-5, and NOTCH1 as independent predictors of HF hospitalization in the CKD population, implicating endothelial dysfunction, angiogenesis, and complement activation [72]. Larger analyses of 4638 proteins identified over 200 HF-associated proteins, including angiopoietin-2, fibulin-5, growth differentiation factor-15 (GDF-15), and SVEP1, with Mendelian randomization supporting causal roles for IGFBP3 and ASGR1 and improved predictive accuracy compared with clinical models (C-statistic 0.790 vs. 0.703) [73]. Similarly, proteomics identified 44 circulating proteins including GOLM1, ANGPT2, GDF-15, EFEMP1, and SVEP1 associated with CKD severity and incident HF, with Mendelian randomization supporting GOLM1 as a causal mediator of myocardial remodeling [74]. SOMAscan profiling of up to 6376 proteins confirmed CKD-associated HF is characterized by upregulation of inflammatory cytokines, extracellular matrix proteins, and stress mediators in both HF with reduced ejection fraction (HFrEF) and HF with preserved ejection fraction (HFpEF) phenotypes [75]. In type 2 diabetes, proteomics identified subtype-specific proteins such as NEFL and CALB1 associated with CKD progression and HF, implicating inflammatory and metabolic pathways [76]. In HFpEF, kidney injury biomarkers including cystatin-C, NGAL, FABP3, and KIM-1 were associated with mortality and HF hospitalization, with proteomics identifying over 500 inflammatory, complement, and fibrotic mediators linking renal injury to cardiac dysfunction [63].

Proteomics has also provided mechanistic insight into AKI and renal recovery. Non-invasive urinary proteomics has demonstrated strong diagnostic and prognostic utility. Capillary electrophoresis–mass spectrometry identified 107 urinary peptides, primarily collagen I and III fragments, reflecting extracellular matrix remodeling, with classifiers achieving 84% sensitivity and 91% specificity for HF despite CKD-related overlap [77]. Larger urinary peptidomic analyses identified classifiers HF2, CAD160, and CKD273 as predictors of HF, coronary artery disease, and CKD progression (HR 2.59, 1.71, and 4.12), reflecting extracellular matrix remodeling and fibrosis. Beyond risk prediction, these classifiers enable molecular stratification and may identify patients more likely to benefit from targeted therapies, supporting their role in precision cardiorenal medicine [78].

#### 3.5.2. Genomic Determinants and Genetic Susceptibility in Cardiorenal Syndrome

Genomic studies have identified multiple genetic determinants contributing to the development and progression of cardiorenal disease through mechanisms involving oxidative stress, neurohormonal activation, inflammation, and metabolic dysregulation. Loss of glutathione S-transferase mu 1, a key enzyme regulating cellular redox balance, represents an important genomic risk factor, with copy number variation associated with significantly increased risks of kidney failure (HR 1.66, 95% CI 1.27–2.17) and HF (HR 1.16, 95% CI 1.04–1.29) over 24.6 years of follow-up in the ARIC cohort. That factor highlights oxidative stress-mediated tubular injury, myocardial remodeling, and fibrosis as shared pathogenic mechanisms [79]. Similarly, genomic variation in the RAAS plays a central role in cardiovascular risk modulation in end-stage kidney disease. The AGTR1 rs5186 polymorphism, identified in the EVOLVE genetic substudy of 1472 hemodialysis patients, was associated with a 25–34% increased risk of cardiovascular events, HF, and mortality, mediated through disruption of microRNA-155-dependent repression and amplification of angiotensin II signaling pathways promoting inflammation, fibrosis, and vascular dysfunction. Additional ancestry-specific associations between ACE polymorphisms and sudden cardiac death further emphasize the importance of RAAS-related genomic variation in cardiorenal risk [80].

Polygenic risk approaches have further clarified the genetic architecture of cardiovascular risk in CKD populations. In the CRIC, a prospective, multicenter study including patients with moderate CKD (eGFR 20–70 mL/min/1.73 m^2^) followed for over a decade, blood pressure genetic risk scores derived from 901 loci were independently associated with a 10–15% increased risk of MI, stroke, and HF, without affecting CKD progression [81]. Similarly, genetic variation in APOL1, particularly the G3 risk variant, was associated with increased cardiovascular mortality (HR ~1.44) and sudden cardiac death (HR ~1.69) in hemodialysis patients, implicating APOL1-mediated inflammation, ion channel dysfunction, and myocardial remodeling as mechanisms linking kidney disease with cardiovascular mortality [82]. Mendelian randomization analyses using large-scale GWAS consortia further demonstrated a causal effect of genetically predicted HF on CKD development (OR 1.12, 95% CI 1.03–1.21), confirming cardiac dysfunction as a primary upstream driver of renal injury through neurohormonal activation and fibrotic signaling pathways [83].

Pharmacogenomic and multi-locus genomic analyses have also identified genetic determinants influencing both cardiovascular and renal outcomes. In the UK Biobank cohort, genetic variants in RYR3, CYP3A5, and ADRA1A were associated with increased risks of both HF and CKD, with CYP3A5 rs776746 homozygotes demonstrating more than a twofold increased CKD risk and RYR3 rs877087 carriers showing increased HF incidence, highlighting the importance of ion channel regulation, drug metabolism, and vascular signaling pathways in patients with CRS [84].

Collectively, these genomic findings establish oxidative stress regulation, neurohormonal signaling, blood pressure regulation, and metabolic genetic pathways as central molecular determinants of CRS, supporting the integration of genomic profiling into precision risk stratification and the identification of novel therapeutic targets.

#### 3.5.3. Inflammatory and Cytokine Signaling Mechanisms in Cardiorenal Pathophysiology

Inflammatory cytokine signaling represents a central molecular mechanism linking kidney dysfunction to HF. IL-6 has emerged as a key inflammatory mediator, with analysis of 3031 CKD patients in the CRIC cohort demonstrating an independent association of IL-6 levels with markedly increased risk of all-cause mortality (HR 4.11) and cardiovascular events (HR 2.52) over 10 years [85]. Complementing these findings, integrative genomics and proteomics analyses using Mendelian randomization identified multiple inflammatory and fibrosis-related proteins as causal mediators of HF, including IL1R1, GDF15, CTSO, ANGPT2, FGF19, TIMP4, COL28A1, TP53, and complement component C5 [86]. IL1R1 emerged as a central cytokine signaling hub mediating inflammatory responses, while GDF15, CTSO, and COL28A1 were implicated in myocardial fibrosis and extracellular matrix remodeling. Complement activation and fibroblast growth factor signaling further highlighted immune activation and metabolic dysregulation as key contributors to HF development.

#### 3.5.4. Metabolomic Alterations and Metabolic Pathway Dysregulation in Cardiorenal Disease

Metabolomics studies have identified lipid and amino acid metabolic dysregulation as central mechanisms in cardiorenal disease progression and therapeutic response. Targeted metabolomics using LC-MS/MS and FIA-MS/MS identified 208 metabolites (like ceramides, sphingomyelins, phosphatidylcholines, and amino acid pathway intermediates) and key biomarkers such as cystine, stearic acid, and sphingomyelin SM C16:0, capable of distinguishing advanced HFpEF among CKD patients. Its diagnostic performance was superior to NT-proBNP [87]. Elevated uraemic toxins including ADMA, SDMA, homocysteine, and creatinine reflected impaired renal clearance and reduced nitric oxide bioavailability, linking renal dysfunction with endothelial impairment and HF progression. Complementary untargeted metabolomics using UPLC-Q-TOF/MS and machine learning identified lysophosphatidylcholine species, decanoylcarnitine, and L-proline as predictors of sacubitril/valsartan response in ESRD patients, with LysoPC accumulation driven by PLA2G4A-mediated phospholipid remodeling promoting inflammation, thrombosis, and myocardial remodeling [88]. These findings establish metabolomics-defined lipid dysregulation, uraemic toxin accumulation, and PLA2G4A-dependent phospholipid metabolism as key molecular mechanisms and therapeutic determinants in CRS.

#### 3.5.5. Multi-Omics Integration and Systems Biology Approaches in Cardiorenal Syndrome

Multi-omics integration combining genomics, transcriptomics, proteomics, and imaging has provided systems-level insight into the molecular architecture of cardiorenal disease and significantly improved risk prediction. In the UK Biobank cohort of 8646 participants, integration of polygenic risk scores with multi-organ imaging phenotypes increased predictive accuracy for HF from AUC 0.63 using genomics alone and 0.68 using imaging alone to 0.79 in combined models, and improved coronary artery disease prediction to AUC 0.81, highlighting the additive value of combining genetic susceptibility with structural organ phenotypes such as cardiac and renal function [89]. Mendelian randomization integrated with transcriptomics further identified causal genomic regulators including PDE5A and GUCY1A3, implicating NO–sGC–cGMP signaling as a protective pathway modulating cardiovascular and renal risk, while genetic proxies targeting PDE5, endothelin receptor, and angiotensinogen pathways were associated with reduced risks of coronary artery disease and CKD, supporting these pathways as therapeutic targets [90]. Comprehensive multi-omics integration incorporating genomics, epigenomics, transcriptomics, and proteomics has identified key regulatory genes including ANAPC4, UNC50, and TPO, and proteins such as HP, FCGR3B, GALNT2, SERPINA1, and FER, which are involved in inflammation, immune activation, and metabolic dysfunction. Additional targets including ACE, SREBF1, PRKG1, COL18A1, and STIM1 implicate oxidative stress, endothelial dysfunction, and vascular remodeling as central mechanisms linking cardiovascular, renal, and metabolic disease [91]. Collectively, these findings demonstrate that multi-omics integration enables identification of causal molecular networks, improves risk stratification beyond single-modality approaches, and provides a comprehensive framework for precision medicine and therapeutic targeting in CRS.

However, the clinical application of genomic and multi-omic approaches remains limited by platform-dependent measurements, batch effects, complex bioinformatic processing, high cost, and limited external validation and standardisation. Their findings may also be influenced by ancestry, age, sex, comorbidities, medication exposure, diet, and sampling conditions, reducing comparability and generalisability across CRS populations [13,14,70,71,89,90,91].

### 3.6. Cytokines and Inflammatory Biomarkers

#### 3.6.1. Overview

Many pro-inflammatory cytokines have been recognized as strong prognostic predictors in patients with CHF through a complex mechanism of activation of various neurohormonal pathways [26]. Both CHF and CKD are states of chronic inflammation with elevated levels of circulating inflammatory mediators, and it has been repeatedly shown that advanced CKD is associated with elevated levels of the same pro-inflammatory cytokines that have been studied in CHF. The elevation of IL-6, TNF-α, IL-1, the IL-33/ST2 axis, and IL-18 in patients with both CKD and CHF may suggest a possible role for these cytokines in CRS [5].

#### 3.6.2. Interleukin-6 (IL-6)

##### Pathophysiology

IL-6 is a pleiotropic cytokine centrally involved in cardiovascular and renal pathophysiology. It is rapidly upregulated following MI and contributes to adverse ventricular remodeling, neurohormonal activation, and systemic inflammation among HF patients [92]. In parallel, IL-6 plays a key role in CKD, where it promotes endothelial dysfunction, oxidative stress, and progressive renal fibrosis [93]. Notably, a combined inflammatory profile including hs-CRP, IL-6, and TNF-α has been strongly associated with adverse lifestyle factors and high risk of CRS [94].

##### Interaction with Renal Function

Patients with CRS have demonstrated greater neurohormonal activation, tubular injury, and myocardial damage compared with HF patients with preserved renal function [18]. Plasma and urinary IL-6 appear to reflect distinct yet complementary components of CRS. In ambulatory HF patients requiring intensive diuretic therapy, plasma IL-6 was independently associated with systemic neurohormonal activation and mortality, whereas urinary IL-6 correlated with renal dysfunction, diuretic resistance, and intrarenal neurohormonal activation, but not with survival [27].

##### Diagnosis and Prognosis

Plasma IL-6 has been shown to independently predict overall and cardiovascular mortality in patients at different stages of CKD [95]. In the CRIC cohort, IL-6 and TNF-α were independently associated with the incidence of atherosclerotic vascular events and death in patients with CKD, and the addition of inflammatory markers and kidney function measures significantly improved risk prediction beyond traditional cardiovascular risk scores [96]. Similarly, in patients with chronic CAD and all stages of chronic CKD, elevated levels of IL-6 are associated with increased risk of MACEs [97]. Likewise, in patients with end-stage kidney disease and established cardiovascular disease, elevated IL-6 predicts incident cardiovascular events [98]. In HF populations, elevated circulating IL-6 levels are consistently associated with mortality and HF hospitalization, often retaining prognostic significance after adjustment for renal function [99]. The aforementioned results underscore the incremental prognostic value of inflammatory profiling in populations with chronic CRS.

In acute CRS, a pronounced systemic inflammatory state characterized by exaggerated elevation of IL-6 levels has been described, approaching the high inflammatory milieu observed in end-stage CKD [100]. Patients with CRS type 1 exhibit increased circulating pro-inflammatory cytokines, primarily IL-6 and IL-18, along with heightened oxidative stress, further supporting the central role of inflammatory activation in acute heart–kidney interactions [28].

Despite its prognostic value, IL-6 interpretation is limited by assay variability, biological fluctuation, and the absence of CRS-specific thresholds. Infection, acute ischaemic injury, obesity, and other systemic inflammatory conditions may also increase IL-6 independently of CRS, limiting its specificity as a standalone cardiorenal biomarker.

#### 3.6.3. Tumour Necrosis Factor-α (TNF-α)

##### Pathophysiology

TNF-α is a potent pro-inflammatory cytokine secreted from both the heart and the kidney tissues in response to ischaemia and reperfusion injury [101]. In the myocardium, TNF-α exerts direct deleterious effects, including down-regulation of sarcoplasmic reticulum proteins, impaired contractility, and induction of cardiomyocyte apoptosis [102]. Those mechanisms may contribute to the progression of CHF. In critically ill patients with acute renal failure, elevated TNF-α levels have been documented alongside other inflammatory mediators [103] and experimental models have demonstrated increased renal TNF-α expression following MI and in CRS settings [104], supporting its role in heart–kidney inflammatory crosstalk.

##### Interaction with Renal Function

A notable characteristic of biomarker application is that worsening renal function itself is associated with progressive increases in circulating TNF-α and its soluble receptors, reflecting sustained immune activation and cytokine upregulation in patients on dialysis [105]. This imbalance between TNF-α and its soluble receptors (sTNFR1 and sTNFR2) complicates interpretation, as elevated levels may reflect renal retention or chronic inflammatory activation rather than specific cardiorenal injury. Thus, despite strong biological plausibility and consistent associations with disease severity, TNF-α remains limited by poor specificity, renal confounding, and lack of validated CRS-specific thresholds.

##### Diagnosis and Prognosis

Clinically, elevated plasma TNF-α has been associated with the development of CRS in patients with end-stage renal disease [106], and patients with CRS type 1 exhibit increased circulating TNF-α and IL-6 levels, along with oxidative stress and tissue injury markers [28]. Furthermore, circulating TNF-α has been shown to provide prognostic information in AHF populations, where pre-existing renal dysfunction remains a strong predictor of mortality [107].

TNF-α measurement is also limited by assay variability and the differing circulating kinetics of free TNF-α and its soluble receptors. Infection, acute ischaemic injury, and other systemic inflammatory conditions may elevate TNF-α independently of CRS, reducing its diagnostic specificity [28,105,106,107].

#### 3.6.4. Interleukin-1β (IL-1β)

##### Pathophysiology

IL-1β is an upstream pro-inflammatory cytokine generated through inflammasome activation and represents an early trigger of downstream inflammatory cascades, including IL-6 signaling [108]. In both HF and CKD, IL-1β contributes to myocardial function suppression, endothelial dysfunction, leukocyte recruitment, and renal inflammatory injury, positioning it as a plausible amplifier of cardiorenal interactions. Experimental data further support increased cardiac and renal inflammasome activation following ischaemic injury, reinforcing the mechanistic link between IL-1β signaling and heart–kidney cross-talk [109].

##### Interaction with Renal Function

In CKD and dialysis, background inflammatory signaling is altered, including changes in IL-1β/TNF-α and their endogenous inhibitors; therefore, circulating IL-1β should be interpreted cautiously as part of a broader renal-inflammatory milieu rather than as a CRS-specific signal [105,110]. In patients with HF and concomitant renal dysfunction, IL-1 pathway activation appears clinically relevant, as IL-1 blockade with anakinra reduced systemic inflammatory activity in a pilot cardiorenal cohort, supporting IL-1-mediated inflammation despite impaired renal function [111].

##### Diagnosis and Prognosis

Clinical evidence directly evaluating IL-1β as a biomarker in CRS is limited, with most data derived from HF cohorts, small mechanistic studies, or post hoc analyses of interventional trials targeting the IL-1 pathway. While IL-1β-related signaling has been linked to adverse cardiovascular outcomes and inflammatory burden, its short half-life, low detectable circulating concentrations, and assay variability limit its reliability as a standalone biomarker [110].

In a pilot study of HF patients with renal dysfunction, IL-1 blockade with anakinra significantly reduced systemic inflammatory markers, supporting a mechanistic role of IL-1-mediated inflammation in cardiorenal interactions [111]. Similarly, results from the CANTOS trial demonstrated that selective IL-1β inhibition with canakinumab reduced rates of MACEs in high-risk atherosclerotic patients with CKD, particularly among those achieving a robust anti-inflammatory response [112]. However, these studies primarily evaluated therapeutic modulation rather than validating IL-1β as a prognostic CRS biomarker, and CRS-specific outcome thresholds remain undefined.

#### 3.6.5. Interleukin-33 (IL-33)/Soluble Suppression of Tumorigenicity-2(sST2)

##### Pathophysiology

sST2 is a circulating member of the IL-1 receptor family that reflects myocardial biomechanical stress and inflammatory activation [29]. It is released predominantly by cardiac fibroblasts and endothelial cells in response to pressure and volume overload and functions as a decoy receptor for IL-33, thereby inhibiting signaling through the membrane-bound ST2L receptor on cardiomyocytes [113]. While IL-33/ST2L signaling exerts cardioprotective effects, including anti-hypertrophic and anti-fibrotic actions, elevated circulating sST2 levels attenuate this protective pathway and are therefore associated with adverse myocardial remodeling and progressive ventricular dysfunction.

##### Interaction with Renal Function

Evidence from HF cohorts supports this relative renal independence. In patients hospitalized with AHF and renal insufficiency, sST2 levels were not significantly affected by the degree of renal dysfunction, in contrast to BNP, which increased markedly with declining eGFR; predischarge sST2 independently predicted short-term mortality and readmission [114]. Similarly, although sST2 demonstrated a weak inverse correlation with eGFR (*p* = −0.16), its prognostic performance for long-term mortality was preserved across renal function levels [115]. In a larger cohort, sST2 showed significantly weaker univariate correlation with eGFR compared with NT-proBNP (r = −0.147 vs. −0.525) and maintained stable diagnostic cutoffs across CKD and non-CKD groups; when combined with NT-proBNP, it significantly improved HF diagnostic accuracy in patients with impaired kidney function [116].

##### Diagnosis and Prognosis

Clinically, sST2 provides incremental prognostic value beyond NPs for predicting HF-related mortality and hospitalization and, importantly, appears minimally influenced by renal function [114]. From a cardiorenal perspective, elevated baseline sST2 levels in advanced HFrEF were independently associated with increased mortality even after accounting for longitudinal eGFR changes, whereas other biomarkers such as soluble urokinase-type plasminogen activator receptor and gal-3 were more closely linked to renal function decline [117]. Moreover, in patients with AMI, sST2 independently predicted the development of CRS type 1 during hospitalization, demonstrating strong discriminative performance (AUC 0.874), and risk stratification was further improved when combined with eGFR and NT-proBNP [118].

Taken together, sST2 provides consistent prognostic information and is relatively less influenced by renal function than several other cardiac biomarkers. Nevertheless, assay-dependent thresholds and elevations caused by systemic inflammation or non-CRS cardiac stress may complicate interpretation, while sST2 primarily reflects cardiac biomechanical stress rather than bidirectional organ injury; consequently, CRS-specific thresholds remain undefined.

#### 3.6.6. Interleukin-18 (IL-18)

##### Pathophysiology

IL-18 is an inflammasome-related cytokine implicated in myocardial injury, vascular inflammation, and progression of renal dysfunction [119]. Beyond serving as a marker of systemic inflammation, it directly stimulates B-type natriuretic peptide synthesis in cardiomyocytes via PI3K–AKT signaling and correlates with BNP levels in AHF, with higher concentrations observed in patients with concomitant renal impairment [120]. These findings provide mechanistic support for a link between inflammasome activation, myocardial stress signaling, and renal impairment.

##### Interaction with Renal Function

In acute CRS, IL-18 appears to reflect tubular injury and inflammatory activation. In a prospective multicenter cohort of patients with acute CRS, urinary IL-18 measured at the time of AKI diagnosis independently predicted progression of AKI and improved risk reclassification beyond clinical models, although its discriminative performance was inferior to urinary angiotensinogen [20]. Similarly, in patients hospitalized for decompensated AHF, urinary IL-18 was only a modest predictor of in-hospital AKI but independently predicted persistent renal impairment at 6 months and was significantly associated with all-cause mortality, suggesting that IL-18 may better reflect sustained tubular injury and long-term cardiorenal risk rather than AKI detection [21].

##### Diagnosis and Prognosis

Inflammatory amplification in CRS type 1 further supports its biological relevance. Patients with CRS1 exhibit significantly higher circulating IL-18 levels compared with AHF patients without AKI, alongside elevations in IL-6, NGAL, and oxidative stress markers [28]. Moreover, IL-18 correlates with caspase-3 and circulating cell-free DNA concentrations, linking inflammasome activation to apoptosis and tubular injury in acute cardiorenal dysfunction [121]. Therapeutically, nicorandil administration in CRS type 1 was associated with significant reductions in IL-18 and other renal injury markers (NGAL, KIM-1), along with improvement in cardiac and renal function. IL-18 may therefore reflect dynamic inflammatory activity in acute CRS [64].

Outside the acute CRS setting, higher urinary IL-18 levels were associated with incident HF in a community-based cohort, although this association was attenuated after adjustment for albuminuria, indicating limited specificity beyond established renal markers [122]. Collectively, IL-18 captures both cardiac stress signaling and tubular injury pathways in CRS. However, its moderate discriminative performance for early AKI and overlap with other renal biomarkers limit its clinical applicability per se, positioning IL-18 as an adjunctive rather than definitive CRS biomarker.

Collectively, cytokine biomarkers are limited by short half-lives, low circulating concentrations, assay variability, and the absence of standardized CRS-specific thresholds. Beyond renal dysfunction, infection, acute ischemic injury, obesity, and other systemic inflammatory conditions may alter cytokine concentrations, reducing their specificity for bidirectional cardiorenal injury and limiting routine cytokine-guided risk stratification.

### 3.7. Galectin-3

#### 3.7.1. Pathophysiology

Gal-3 is a 30 kDa β-galactoside-binding lectin encoded by LGALS3 and predominantly expressed in macrophages, endothelial and epithelial cells, and renal tubular tissue [30]. It functions as a mediator of inflammation-driven fibrosis by promoting macrophage activation, fibroblast proliferation, and extracellular matrix deposition, thereby contributing to myocardial remodeling and renal structural injury [31]. Circulating concentrations typically range from 10 to 13 ng/mL in healthy individuals and increase in HF, correlating with disease severity and renal impairment. Positioned within the cardiac–renal–metabolic axis, Gal-3 integrates inflammatory and profibrotic pathways linking HF, CKD, and diabetes. Clinical data support the association of elevated serum levels with adverse outcomes in CHF and increased all-cause mortality in non-dialysis CKD (HR per unit 1.22, 95% CI 1.05–1.41; ≥6 ng/mL HR 2.66, 95% CI 1.68–4.23) [123].

#### 3.7.2. Interaction with Renal Function

Across CKD populations, gal-3 demonstrates consistent association with renal progression, while its association with mortality is inconsistent. In CHF outpatients, gal-3 may independently predict 1-year worsening renal function (OR 1.09) [124]. A meta-analysis of 5226 CKD patients showed pooled HR 1.38 for all-cause mortality and 1.05 for cardiovascular events; however, in haemodialysis subgroups, mortality association was not significant (HR 1.17) [125].

Previous studies recruiting hemodialysis patients have shown conflicting results regarding the association of gal-3 with all-cause mortality and cerebrocardiovascular events [124,126].

#### 3.7.3. Diagnosis and Prognosis

In community-based cohorts, gal-3 predicts incident cardiac or renal failure. In 9148 ARIC participants without baseline CKD or HF, higher gal-3 independently predicted incident CKD over 16 years (Q4 vs. Q1 HR 1.75, 95% CI 1.49–2.06), persisting after adjustment for eGFR, UACR, cTnT, and NT-proBNP [127]. In 2477 Framingham Offspring participants, increases in Gal-3 during 10-year monitoring independently predicted incident HF (HR 1.39 per SD), CVD (HR 1.29), and all-cause mortality (HR 1.30). Persistently high or rising levels were associated with the greatest risk [128]. In 1320 patients with type 2 diabetes (eGFR ≥ 30 mL/min/1.73 m^2^), gal-3 independently predicted doubling of serum creatinine (HR 1.19 per ng/mL) and incident macroalbuminuria (HR 1.20), with the highest quartile associated with fourfold likelihood of renal function loss and threefold risk of macroalbuminuria occurrence [129].

In CHF, gal-3 reflects severity and independently predicts outcomes in selected settings. In DEAL-HF (n = 232, predominantly NYHA III), gal-3 independently predicted 6.5-year mortality (HR per SD 1.24, 95% CI 1.03–1.50) beyond eGFR and NT-proBNP [30]. In 190 patients with prior MI and CHF, Gal-3 > 21 ng/mL independently predicted 26-month mortality alongside cystatin-C > 2800 pg/mL [130]. In Val-HeFT trial (n = 1650), the baseline gal-3 correlated strongly with renal dysfunction (eGFR explaining 20% variability) but lost independent prognostic value after full adjustment. However, serial increases at 4 and 12 months independently predicted mortality and HF events, and valsartan reduced HF hospitalisations only in patients with baseline gal-3 below 16.2 ng/mL [131]. In 151 HFrEF patients in the PROTECT study, gal-3 > 20 ng/mL independently predicted cardiovascular events and renal dysfunction, yet intensified mineralocorticoid receptor antagonist therapy did not counteract the adverse outcomes, indicating limited therapeutic guidance [132].

In RELAX trial (n = 216), among diabetic patients those with HFpEF had significantly higher gal-3 (15.5 vs. 13.1 ng/mL, *p* < 0.001) with a profibrotic-inflammatory profile and greater risk for cardiorenal cause of hospitalization [133]. In two other studies recruiting HFpEF patients, LogGal-3 ≥ 1.3 independently predicted 6-month death/cardiovascular rehospitalization after acute decompensation and adjustment for renal dysfunction [134], while gal-3 independently predicted HF hospitalization or cardiovascular death (n = 154 patients) beyond MAGGIC score, BNP, age, and eGFR, despite interaction with renal function [135].

In AHF, gal-3 closely tracks renal injury and short-term risk. In DECIDE trial (n = 201) the upper quartile of gal-3 on admission predicted 30-day events, particularly within a cardiorenal phenotype [136]. In AKINESIS trial (n = 790), gal-3 > 25.9 ng/mL was associated with lower eGFR, higher uNGAL and hs-cTnI, AKI, and independently predicted 1-year mortality (HR 1.61 per doubling of gal-3 levels) but not rehospitalization [137]. In 1201 hospitalized patients with AHF, a prognostic cutoff value of 31.5 ng/mL was confined to eGFR < 60 mL/min/1.73 m^2^ [138].

In advanced HFrEF (REVIVAL trial, n = 309), baseline gal-3 independently predicted 2-year eGFR decline (b −0.14 per SD), but after controlling for longitudinal eGFR change in joint models, gal-3 was not independently associated with mortality, unlike ST2 and NT-proBNP [117]. In contrast, in BIOSTAT-CHF trial (n = 2180; validation n = 1703), gal-3 clustered with renal markers and independently predicted only all-cause mortality after adjustment, underscoring its cardiorenal phenotype [139]. Finally, after heart transplantation (n = 62), gal-3 remained elevated in 56% at 1 year, strongly inversely correlated with eGFR and not with myocardial fibrosis, reinforcing its systemic, renally influenced nature [140].

The apparently conflicting findings regarding gal-3 may be explained by differences in patient populations, HF phenotype, severity of renal dysfunction, study endpoints, and whether baseline or serial measurements were evaluated. In some HF and CKD cohorts, gal-3 independently predicted mortality, hospitalization, or renal deterioration [30,125,127,128,129,130,132,134,135,136,137]. However, in Val-HeFT and REVIVAL, its independent association with mortality was attenuated or lost after adjustment for renal function, whereas serial increases remained prognostically informative [115,134]. Similarly, results in hemodialysis populations were inconsistent, and the association with mortality was not significant in the hemodialysis subgroup of a meta-analysis [124,125,126]. These differences suggest that gal-3 may function primarily as a marker of a systemic cardiorenal and fibrotic phenotype, strongly influenced by renal function, rather than as a consistently independent cardiac prognostic biomarker.

Gal-3 interpretation is limited by assay-dependent thresholds, heterogeneous study cut-offs, and uncertainty regarding clinically meaningful serial changes. Beyond renal dysfunction, diabetes, systemic inflammation, cardiac remodeling, and other fibrotic conditions may increase gal-3 concentrations, limiting its specificity for bidirectional cardiorenal injury and its use as a standalone CRS biomarker.

### 3.8. Cardiac-to-Renal Influence and Renal Biomarker Response

The cardiac-to-renal direction of CRS is particularly evident in acute decompensated HF, where renal hypoperfusion and venous congestion may cause early deterioration in renal function [19]. Cystatin C may detect this deterioration before serum creatinine increases and may therefore identify subclinical renal dysfunction during cardiac decompensation [19]. Urinary IL-18 has also been associated with AKI progression, persistent renal impairment, and mortality in patients with acute CRS [20,21]. In CHF, galectin-3 has been associated with worsening renal function and renal dysfunction [124,132], although its prognostic interpretation is limited by its close relationship with renal function [131,139,140]. Collectively, these findings illustrate how cardiac dysfunction may produce measurable changes in renal and cardiorenal biomarkers [19,20,21,124,131,132,139,140].

### 3.9. Systemic Pathway-Based Interpretation of Biomarkers in CRS

Beyond organ-specific biomarkers, CRS can also be interpreted through systemic pathways shared by HF and CKD. Chronic inflammation is reflected by elevated IL-6, TNF-α, IL-1 and IL-18, together with alterations in the IL-33/sST2 axis, across both conditions [26,101]. Fibrotic and remodeling activity is reflected by gal-3, which contributes to myocardial remodeling and renal structural injury, and by sST2, which reflects myocardial biomechanical stress and adverse remodeling [29,30,31,113]. Oxidative stress represents another common mechanism, supported by GSTM1-related redox dysregulation and multi-omic findings implicating oxidative stress, endothelial dysfunction, and vascular remodeling in cardiorenal disease [79,91]. Proteomic studies have also identified concurrent alterations in inflammatory cytokines, extracellular matrix proteins, and stress mediators in CKD-associated HF, supporting a systemic, pathway-based and multi-marker interpretation of CRS [63,70,71,75].

### 3.10. Diabetes Mellitus as a Systemic Model of CRS

Diabetes mellitus represents a characteristic example of type 5 CRS because it may concurrently affect cardiac and renal function through a common systemic disease process [1,2,3,4,7,8]. In patients with type 2 diabetes, gal-3 has been associated with the progression of nephropathy, while diabetic patients with HFpEF have demonstrated higher gal-3 concentrations and a more pronounced profibrotic-inflammatory profile [129,133]. Studies involving patients with diabetes and diabetic kidney disease have also evaluated NT-proBNP, hs-cTnT, GDF-15, TNFR-1, TNFR-2, and KIM-1 as indicators of cardiac stress, systemic inflammation, and renal or tubular injury [65,141,142,143]. Diabetes therefore illustrates the difficulty of establishing cause-and-effect relationships in CRS, because changes in circulating biomarkers may reflect the systemic metabolic disease, cardiac dysfunction, renal injury, or the effects of treatment rather than a single organ-specific process [65,141,142,143,144].

## 4. The Therapeutic Modulation of Biomarkers in CRS: Novel Treatment with Sodium-Glucose Cotransporter-2 Inhibitors (SGLT2i)

Sodium-glucose cotransporter-2 inhibitors (SGLT2i) play a key role in the management of cardiorenal disease, demonstrating important benefits in HF and CKD populations independent of the presence of diabetes [141,144,145]. In CRS, SGLT2i may exert multifunctional mechanisms: reduction in intraglomerular pressure, increased osmotic diuresis and natriuresis, improvement of cardiac energetics and thus kidney perfusion, and reduction of inflammation and fibrosis, leading to clinical benefits [144,145]. Landmark trials of SGLT2i, such as EMPEROR, DECLARE-TIMI 58, CANVAS, and CREDENCE, have shown the favourable modification of cardiorenal pathophysiology via biomarker changes [65,141,142,146,147]. Those related biomarkers (NPs, cardiac injury markers, renal and tubular injury biomarkers, inflammatory mediators, and fibrosis/metabolic markers) not only provide a better understanding of the underlying pathophysiological pathways but also can be used for CRS monitoring.

### 4.1. Effects of SGLT2i on NPs and Other Cardiac Biomarkers

NPs, NT-proBNP and BNP, are pivotal biomarkers for HF diagnosis, risk stratification, and therapeutic monitoring [148]. However, in patients with CRS, NP levels are often elevated due to reduced renal clearance and chronic volume expansion, confusing the interpretation of their levels [143]. Furthermore, Neuen et al. examined the complex interplay between NT-proBNP, kidney function, and clinical outcomes in patients with HFpEF [49]. Despite higher baseline levels of NT-proBNP, they maintained their prognostic significance across the range of eGFR [49]. The EMPEROR-Reduced trial revealed the strong impact of SGLT2i therapy on NT-proBNP in patients with HFrEF. In particular, empagliflozin administration significantly reduced NT-proBNP levels compared to placebo, in parallel with improved clinical outcomes (lower rates of cardiovascular death and HF hospitalisation) [146]. Notably, approximately half of enrolled patients had eGFR < 60 mL/min/1.73 m^2^, and benefits were consistent across CKD subgroups. Analysis of NT-proBNP data from the CREDENCE and CANVAS trials by Bayes-Genis et al. demonstrated that in patients with type 2 diabetes and CRS, canagliflozin significantly reduced NT-proBNP levels from baseline, and the magnitude of the reduction was associated with the treatment period. Most importantly, the NT-proBNP reduction was associated with lower rates of HF hospitalization and cardiovascular death, highlighting the role of NPs in prognosis and therapy monitoring [143]. SGLT2 inhibitors may lower NT-proBNP via their mild natriuretic and osmotic diuretic action which can promote decongestion and reduce circulating volume. At the same time, reduced neurohormonal drive and more efficient renal sodium and water handling may support renal perfusion. These changes provide a link between SGLT2 inhibition and reduced ventricular stress, aligning with the observed downward shift in NT-proBNP and the cardiorenal benefits reported in clinical studies.

In the CREDENCE study, multiple cardiorenal biomarkers including NT-proBNP, hs-TnT, GDF-15, and insulin-like growth factor binding protein-7 (IGFBP7) were evaluated by Januzzi et al. Canagliflozin significantly reduced NT-proBNP and hs-TnT levels compared to placebo, suggesting additional attenuation of myocardial injury. Moreover, canagliflozin treatment decreased GDF-15, a stress-responsive cytokine involved in inflammation, metabolism, and tissue repair, while IGFBP7 levels—a marker of cardiorenal pathophysiology—remained stable [141]. The latter correlates with increased risk of cardiovascular death and HF hospitalisation across the ejection fraction spectrum. EMPEROR-Reduced and EMPEROR-Preserved trials demonstrated that empagliflozin did not significantly reduce IGFBP7 levels. Hence, the beneficial mechanisms of SGLT2i do not seem to involve the IGFBP7 pathway [149]. Sen et al. examined the role of circulating GDF-15 in the CANVAS trial. Elevated baseline GDF-15 was strongly associated with cardiovascular events, HF hospitalisation, and renal outcomes. Canagliflozin treatment reduced GDF-15 levels, with early changes in GDF-15 showing an association with subsequent clinical outcomes [142].

These biomarker reductions should not be interpreted as direct drug effects. Lower hs-cTnT and NT-proBNP may instead reflect reduced myocardial injury, ventricular stress, congestion, and inflammation resulting from the broader cardiorenal effects of SGLT2 inhibition [141,143,144,145,146,150,151,152,153].

### 4.2. Effects of SGLT2i on Renal and Tubular Injury Biomarkers

Traditional markers of renal function, such as serum creatinine and eGFR, have important limitations in patients receiving SGLT2i, as these drugs induce an early, reversible decline in eGFR due to haemodynamic effects on intraglomerular pressure rather than true nephrotoxicity [145]. Novel tubular injury biomarkers offer the potential to distinguish functional haemodynamic changes from true kidney injury. Sen et al. examined the effects of canagliflozin (CANVAS trial) on plasma inflammation-related biomarkers like tumour necrosis factor receptors 1 and 2 (TNFR-1/2), indices of diabetic kidney disease progression, and KIM-1, a specific marker of proximal tubular injury. TNFR-1 and TNFR-2 levels also decreased modestly with canagliflozin treatment. Importantly, the early reductions in TNFR-1 and TNFR-2 were independently associated with lower risk of kidney disease progression, suggesting these biomarkers may serve as indicators of pharmaceutical renoprotection. Canagliflozin significantly reduced KIM-1 concentrations compared to placebo, indicating lessening of tubular injury despite the eGFR dip. Those findings indicate that SGLT2i-induced eGFR changes reflect beneficial haemodynamic resetting rather than nephrotoxicity [65]. In addition to this, Kugathasan et al. examined the effects of empagliflozin treatment on multiple markers of cardiorenal injury and inflammation in patients with type 1 diabetes [66].

Finally, Thiele et al. examined empagliflozin in patients who were hospitalized with acute decompensated HF—focusing on biomarkers of AKI. Although there was a theoretical concern that the natriuretic effect of SGLT2 inhibition could worsen renal function in the acute phase, empagliflozin was associated with lower tubular injury markers than placebo and did not increase the incidence of AKI [67]. Taken together, these data suggest that SGLT2i may confer renoprotection in both the chronic and acute phases [67].

### 4.3. Effects of SGLT2i on Inflammatory Biomarkers and Gal-3

Chronic, subclinical inflammation plays a key role in both HF and CKD progression and adverse outcomes. SGLT2i have demonstrated anti-inflammatory effects across multiple pathways, which may contribute to their cardiorenal benefits. IL-6 is a key pro-inflammatory cytokine implicated in cardiovascular and renal pathophysiology. A systematic review and meta-analysis of randomized controlled trials conducted by Gohari et al. examined the effects of SGLT2i on IL-6 concentrations [150]. The analysis revealed that SGLT2i therapy was associated with significant reductions in IL-6 levels across the included patient populations compared with control groups. SGLT2i may reduce IL-6 levels by modulating AMPK activation, enhancing autophagy in immune cells, and shifting macrophage polarization—resulting in attenuation of pro-inflammatory cytokine production [150]. Beyond IL-6, SGLT2i have also been associated with lower levels of other inflammatory mediators, including TNF-α, CRP, and several adipocytokines. Importantly, these anti-inflammatory effects seem to translate into less inflammatory cell infiltration in both cardiac and renal tissue [151]. A systematic review by Bray et al. explored the effects of SGLT2 inhibitors on inflammatory and oxidative stress biomarkers and found overall heterogeneous but consistent reductions in CRP and several pro-inflammatory cytokines [152]. The NLRP3 inflammasome has emerged as a critical mediator of cardiorenal inflammation. Kounatidis et al. revealed that SGLT2i affect NLRP3 inflammasome activation, reducing the downstream release of IL-1β and IL-18 in CRS [153].

Gal-3, a β-galactoside-binding lectin involved in fibrosis and inflammation, has emerged as a promising biomarker in cardiorenal disease. In the DECLARE-TIMI 58 trial, higher baseline gal-3 correlated with worse eGFR and steeper renal decline. Dapagliflozin did not substantially lower gal-3 concentrations, yet it remained reno-protective. Thus, current data do not support any interaction between SGLT2i and gal-3-associated fibrotic pathways [147].

### 4.4. Effects of SGLT2i on Fibrosis Haematologic and Metabolic Biomarkers

Myocardial and renal fibrosis are key contributors to the progression of CRS, and circulating biomarkers of collagen turnover can provide insight into ongoing extracellular matrix remodeling. Based on EMPEROR trials, empagliflozin induced modest but meaningful reductions in circulating markers linked to collagen synthesis, particularly PICP and PRO-C3. Those changes were observed across HF phenotypes, supporting the possibility that SGLT2 inhibition may influence fibrotic processes, not necessarily directly, in HF patients [154]. Importantly, accumulated evidence indicates that the rise in haematocrit with SGLT2i is at least partly driven by enhanced erythropoiesis. This effect is thought to result from improved renal oxygen balance and more efficient erythropoietin production. By increasing red blood cell mass and oxygen-carrying capacity, this mechanism may improve tissue oxygen delivery and contribute to the broader cardioprotective profile observed with SGLT2i [155]. Finally, iron metabolism is closely linked to erythropoiesis and has been considered in the context of SGLT2i therapy. Analyses of canagliflozin and other SGLT2 inhibitors suggest that increases in haemoglobin may be accompanied by reductions in ferritin and transferrin saturation, consistent with greater utilization of iron stores to support erythropoiesis. Importantly, the cardiorenal benefits of SGLT2 inhibition appear to be maintained across different baseline iron states, and increases in haemoglobin have been shown to correlate with treatment benefit in mediation analyses [155].

### 4.5. Clinical Implications and Future Directions

The biomarker data reviewed here have important clinical implications for the management of CRS with SGLT2i. Across studies, changes in NPs, cardiac injury markers, and indices of tubular stress have been linked with organ-protective effects [65,67,141,146]. Understanding these biomarker trajectories can also support clinical interpretation during treatment. In HF populations, reductions in NT-proBNP may serve as indicators of therapeutic response, although the magnitude of change varies among individuals [143,146]. Biomarkers may also help refine risk stratification. Patients with CRS and higher baseline NT-proBNP, hs-cTnT, or inflammatory burden appear to derive greater absolute benefit from treatment, supporting risk-based prescribing approaches [141,142]. Several areas warrant further investigation. The role of emerging biomarkers such as sST2, IGFBP7, and GDF-15 as treatment selection or monitoring tools requires prospective validation [66,142,149]. The optimal timing and frequency of biomarker assessment during SGLT2i therapy also remain to be defined, and whether biomarker-guided therapeutic strategies can improve outcomes represents an important research question. Overall, SGLT2 inhibitors demonstrate favorable effects across multiple biomarker domains relevant to CRS. These biomarker changes provide mechanistic insight into SGLT2 inhibitor-mediated cardiac and renal protection and may offer practical tools for monitoring therapy in patients with concomitant HF and CKD. As our understanding of cardiorenal biomarker biology evolves, biomarker-guided approaches may further optimize the use of SGLT2 inhibitors in this high-risk population.

## 5. Conclusions

The results of the present comprehensive review outline a significant but challenging role of biomarkers in diagnosis and risk stratification of patients with CRS. A growing body of evidence has shed light on the involvement of cTn and NPs in patients with concomitant HF and CKD. However, the absence of cut-off values and the high influence of kidney dysfunction on absolute values have previously questioned the use of cTn and NPs in CRS. Other emerging biomarkers such as cystatin C and ILs, as well as proteomic approaches, remain under investigation, and they mainly reflect the inflammatory burden and the amount of kidney dysfunction and have limited prognostic value. In parallel, alterations in biomarker levels may help elucidate the proposed beneficial mechanisms of SGLT2i, but they cannot entirely explain the clinical improvement of cardiorenal function and favourable prognosis. Future research may highlight the clinical application of biomarkers in this growing population of patients with CRS.

## Figures and Tables

**Table 1:** Application and clinical interpretation of biomarkers in CRS.

Biomarker	Application in Cardiorenal Syndrome	Interpretation (Diagnosis—Prognosis)
Cardiac Troponins (hs-cTnT/hs-cTnI)	Detection of myocardial injury ACS: serial measurements within 6 h Acute CRS: serial measurements within 6 h Chronic CRS: baseline and discharge measurements for risk stratification	Values above the 99th percentile indicate myocardial injury Association with higher cardiovascular mortality and HF hospitalization risk Baseline elevation (when eGFR < 60) should be considered with caution Δ > 50% change indicates acute myocardial injury and increased short-term adverse outcome risk Persistent elevation reflects ongoing myocardial stress and worse long-term prognosis
Natriuretic Peptides (BNP, NT-proBNP)	Absolute values for HF diagnosis Acute CRS: diagnosis of AHF and guide decongestion therapy Chronic CRS: risk stratification and monitoring of treatment response CKD and age-adjusted cutoffs required due to baseline elevation	Exaggerated elevated levels reflect cardiac wall stress and volume overload Association with increased risk of hospitalization and mortality in CRS ≥30% reduction during therapy suggests effective treatment and improved prognosis
Cystatin C	Absolute measurement for kidney function assessment Acute CRS: May rise before creatinine elevation Chronic CRS: Useful for more accurate eGFR estimation. Combined with creatinine improves eGFR estimation Less influenced by muscle mass vs. creatinine	Elevated levels associated with higher cardiovascular and renal event risk Early rise supports AKI diagnosis and predicts worse renal recovery outcomes Better long-term cardiovascular risk prediction compared with creatinine alone
Genomics/Multi-omics Biomarkers	Research-based risk stratification and phenotyping tools Acute CRS: Limited clinical utility Chronic CRS: Limited clinical utility	No standardized diagnostic cut-offs Identification of CRS subtypes Multi-marker panels with better prediction of adverse cardiorenal outcomes of CRS
Cytokines	Baseline inflammatory risk profiling and monitoring of inflammatory burden Acute CRS: elevation during HF decompensation Chronic CRS: identification of inflammatory disease phenotype	Cautious interpretation in CKD Elevated CRP and cytokines indicate systemic inflammatory activity and increased HF progression and mortality risk Higher GDF-15 and TNFR levels are associated with increased cardiorenal event risk
Galectin-3	Absolute prognostic marker of fibrosis and remodeling Elevated in both HF and CKD Acute CRS: limited diagnostic utility Chronic CRS: prognostic value	Association with higher risk of HF progression and mortality Incremental prognostic value when combined with NPs

Abbreviations: ACS, acute coronary syndrome; AKI, acute kidney injury; BNP, B-type natriuretic peptide; CKD, chronic kidney disease; CRP, C-reactive protein; CRS, cardiorenal syndrome; eGFR, estimated glomerular filtration rate; GDF-15, growth differentiation factor 15; HF, heart failure; hs-cTnI, high-sensitivity cardiac troponin I; hs-cTnT, high-sensitivity cardiac troponin T; NPs, Natriuretic Peptides; NT-proBNP, N-terminal pro–B-type natriuretic peptide; TNFR, tumour necrosis factor receptor; Δ, change.

## Data Availability

No new data were created or analysed in this study. Data sharing is not applicable to this article.

## Abbreviations

The following abbreviations are used in this manuscript:

ACS	acute coronary syndrome
ADHF	acute decompensated heart failure
ADMA	asymmetric dimethylarginine
AHF	acute heart failure
AKI	acute kidney injury
AMI	acute myocardial infarction
AUC	area under the curve
BNP	B-type natriuretic peptide
BP	blood pressure
CAD	coronary artery disease
CKD	chronic kidney disease
CHF	chronic heart failure
CRIC	Chronic Renal Insufficiency Cohort
CRP	C-reactive protein
CRS	cardiorenal syndrome
cTn	cardiac troponin
cTnI	cardiac troponin I
cTnT	cardiac troponin T
eGFR	estimated glomerular filtration rate
ESRD	end-stage renal disease
FGF23	fibroblast growth factor 23
Gal-3	galectin-3
GDF-15	growth differentiation factor 15
GWAS	genome-wide association study
HF	heart failure
HFpEF	heart failure with preserved ejection fraction
HFrEF	heart failure with reduced ejection fraction
HR	hazard ratio
hs-CRP	high-sensitivity C-reactive protein
hs-cTnI	high-sensitivity cardiac troponin I
hs-cTnT	high-sensitivity cardiac troponin T
IL	Interleukin
IL-1β	interleukin-1β
IL-6	interleukin-6
IL-18	interleukin-18
IL-33	interleukin-33
IGFBP7	insulin-like growth factor binding protein-7
KIM-1	kidney injury molecule-1
LC-MS/MS	liquid chromatography–tandem mass spectrometry
LRG1	leucine-rich alpha-2-glycoprotein
MACEs	Major adverse cardiovascular events
MI	myocardial infarction
NGAL	neutrophil gelatinase-associated lipocalin
NPs	natriuretic peptides
NT-proBNP	N-terminal pro-B-type natriuretic peptide
PTH	Parathormone
RAAS	renin–angiotensin–aldosterone system
ROC	receiver-operating characteristic
SD	standard deviation
SDMA	symmetric dimethylarginine
SGLT2i	sodium-glucose cotransporter-2 inhibitors
sST2	soluble suppression of tumorigenicity-2
TNF-α	tumour necrosis factor-α
TNFR	tumour necrosis factor receptor
